# Developing a real-time electronic symptom monitoring system for patients after discharge following cancer-related surgery

**DOI:** 10.1186/s12885-019-5657-6

**Published:** 2019-05-17

**Authors:** Kerry N. L. Avery, Hollie S. Richards, Amanda Portal, Trudy Reed, Ruth Harding, Robert Carter, Leon Bamforth, Kate Absolom, Elaine O’Connell Francischetto, Galina Velikova, Jane M. Blazeby

**Affiliations:** 1 Medical Research Council ConDuCT-II Hub for Trials Methodology Research, National Institute for Health Research Bristol Biomedical Research Centre, Bristol Centre for Surgical Research, Bristol Medical School, Population Health Sciences, University of Bristol, 39 Whatley Road, Bristol, BS8 2PS UK; 2 Medical Research Council ConDuCT-II Hub for Trials Methodology Research, Bristol Centre for Surgical Research, Bristol Medical School, Population Health Sciences, University of Bristol, 39 Whatley Road, Bristol, BS8 2PS UK; 3 Division of Surgery, University Hospitals Bristol NHS Foundation Trust, Bristol, BS2 8HW UK; 4 Section of Patient-Centred Outcomes Research, Patient Reported Outcomes Group, Leeds Institute of Medical Research at St James’s, University of Leeds, St James’s Hospital, Leeds, LS9 7TF UK; 5 NIHR CLAHRC West Midlands Chronic Disease Theme, Institute of Applied Health Research, University of Birmingham, Birmingham, B15 2TT UK

**Keywords:** Adverse effects, Patient-reported outcome measures, Electronic, Electronic health records, Internet, Neoplasms, Operative, Outcome assessment, patient, Self-management, Surgical procedures

## Abstract

**Background:**

Patients undergoing major cancer surgery frequently require post-acute care for complications and adverse effects. Enhanced recovery after surgery programmes mean that patients are increasingly discharged home earlier. Symptom/complication detection post-discharge is sub-optimal. Systematic patient monitoring post-discharge following surgery may be optimally achieved through routine electronic patient-reported outcome (ePRO) data capture. ePRO systems that employ clinical algorithms to guide management of patients and automatically alert clinicians of clinically-concerning symptoms can improve patient outcomes and decrease hospital admissions. ePRO systems that provide individually-tailored self-management advice and integrate live ePRO data into electronic health records (EHR) may also advance personalised health and patient-centred care. This study aims to develop a hospital EHR-integrated ePRO system to improve detection and management of complications post-discharge following cancer-related surgery.

**Methods:**

The ePRO system was developed in two phases: (1) Development of a web-based ePRO symptom-report from validated European Organisation for Research and Treatment of Cancer (EORTC) questionnaires, clinical opinion and patient interviews, followed by hospital EHR integration; (2) Development of clinical algorithms triggering symptom severity-dependent patient advice and clinician alerts from: (i) prospectively-collected patient-completed ePRO symptom-report data; (ii) stakeholder meetings; (iii) patient interviews. Patient advice was developed from: (i) clinician-patient telephone consultations and patient interviews; (ii) review of hospital patient information leaflets (PIL) and patient support websites.

**Results:**

Phase 1, including interviews with 18 patients, identified 35 symptom-report items. In phase 2, 130/300 (43%) screened patients were eligible. 61 (47%) consented to participate and 59 (97%) provided 444 complete self-reports. Stakeholder meetings (9 clinicians, 1 patient/public representative) and patient interviews (*n* = 66) refined advice/alert accuracy. 15 telephone consultations, 7 patient interviews and review of 28 PILs and 3 patient support websites identified 4 themes to inform self-management advice. Comparisons between ePRO symptom-report data, telephone consultations and clinical events/outcomes (*n* = 27 patients) further refined clinical algorithms.

**Conclusions:**

A hospital EHR-integrated ePRO system that alerts clinicians and provides patient self-management advice has been developed to improve the detection and management of problems and complications after discharge following surgery. An ongoing pilot study will inform a multicentre randomised trial to evaluate the effectiveness of the ePRO system compared to usual care.

## Background

Surgery may cure or alleviate cancer but is associated with significant complications and adverse effects (AEs). Up to 30% of patients undergoing major abdominal surgery for oesophageal cancer experience acute or long-term complications, such as infection or the need for further intervention [1], and as many as 45% of patients who have undergone major cancer surgery require post-acute care, such as hospital readmission or community care [2–5]. With more widespread implementation of enhanced recovery after surgery (ERAS) programmes, patients are discharged home increasingly earlier. Detection of complications once at home, however, requires patients to distinguish between symptoms that are typical of recovery (such as expected levels of pain in the first few days) and those that are clinically concerning (such as a change in or worsening of pain). Uncertainty about the significance of symptoms and concerns about self-monitoring and self-care can be worrying and burdensome for patients, their family and caregivers [6], and can delay patients receiving treatment [7–9]. Late detection and treatment of complications can lead to poor patient outcomes [10], impaired quality of life and increased emergency department admissions, with up to 25% of patients being readmitted to hospital within 30 days of major cancer surgery [11, 12].

Prompt identification of complications and AEs after patients are discharged from hospital following surgery for cancer is critical to improve patient safety, outcomes and experiences and enable healthcare professionals (HCP) to plan appropriate care [13–17]. Telephone calls from healthcare providers have been shown to effectively reduce symptoms in cancer patients [18] but allocating HCP time to make patient calls can be cost prohibitive [19]. Instead, systematic monitoring may be optimally achieved through the routine capture of electronic patient-reported outcome data (ePRO), their automatic scoring and the real-time communication of this data to clinicians after hospital discharge. A systematic review of 24 controlled trials concluded that the routine use of PRO measures (PROMs) enhances consultations and may offer improved symptom control and patient satisfaction [20]. However, only two of the 11 electronic systems included were delivered in the patients’ home setting.

Specific features of ePRO systems may provide added benefit. Firstly, the use of clinical algorithms to evaluate patient-reported symptom data and alert healthcare providers when severity reaches a pre-determined threshold can help improve patient outcomes [15, 19]. Basch et al. [15] conducted a large randomised controlled trial (RCT) comparing a web-based Symptom Tracking and Reporting (STAR) ePRO system versus usual care in 766 patients receiving chemotherapy for metastatic solid tumours in the United States, in which severe or worsening symptoms triggered an email alert to a clinical nurse. Findings indicated that the routine and real-time reporting of PROs during cancer surveillance can reduce hospital readmissions and improve patients’ survival and health-related quality of life (HRQL) measured using the EuroQol EQ-5D Index [15]. This study was, however, conducted in a single tertiary referral centre, limiting its generalisability and HRQL was not assessed at follow-up for nearly a third of patients due to attrition resulting from death or discontinuation of treatment. Furthermore, use of a generic PROM measuring only broad aspects of health did not provide insight into the extent to which specific symptoms were improved by symptom reporting.

Patient safety and care may be further enhanced if ePRO are embedded within systems with the functionality to provide patients with individually-tailored advice to support at-home symptom self-management [21]. In a multicentre placebo-controlled RCT of 173 patients conducted in the Netherlands, van der Meij et al. showed how an eHealth intervention that included personalised advice promoted a faster return to normal activities compared to usual care for patients undergoing surgery for benign conditions [22]. Provision of tailored advice is, however, not a common feature of ePRO systems, with many generating PROM data only for clinician review [20, 23]. Finally, making routinely collected ePRO data available to clinicians through its integration into patients’ electronic health records (EHR) has the potential to advance precision health and patient-centred care [24] by enabling ePRO data to be considered alongside other clinical data to better plan appropriate care, to inform patient-clinician encounters and to improve shared clinical decision-making [25]. Integration of ePRO data into patients’ EHR is, however, rare, likely because it is challenging, requiring resources, familiarisation and synchronisation with local information technology (IT) systems, adherence to data security regulations and user training [20, 25].

Several of the ePRO systems developed to date are intended for the oncology setting [26–28]. Most, however, are targeted to patients undergoing chemotherapy, including the STAR system for patients with breast, genitourinary, gynaecological or lung cancer in the US [26] and the ASyMS system for patients with lung, breast and colorectal cancer in the UK [27]. Comparably, few ePRO systems have been developed specifically for patients undergoing surgery for cancer. SIS.NET, for example, is a web-based system developed in the US to improve follow-up care in patients undergoing surgery, radiotherapy, chemotherapy or experimental therapies for breast cancer [28]. Patients randomised to SIS.NET completed regular online health questionnaires with remote review by a nurse practitioner and attended 3 clinic appointments over 18 months. Depending on patient responses, the questionnaire generated automated referrals to support resources at a local cancer centre. Findings from 102 patients showed that the system improved the quality and efficiency of follow-up care, with SIS.NET patients reporting more new or changed symptoms than patients receiving standard care. The symptom questionnaire reports were not, however, integrated within routine hospital EHR. The exact number of patients who received surgery is, also, unknown. To the best of our knowledge, an ePRO system for patients undergoing surgery for cancer with the functionality to integrate with hospital EHR and apply clinical algorithms to alert clinicians and provide individually-tailored self-management advice to guide patient management is lacking [19, 20, 29]. Here, we describe the development of a hospital EHR-integrated ePRO system to improve the detection of complications and AEs after discharge following cancer-related surgery, illustrated in the first instance in the example context of cancer-related major abdominal surgery.

## Methods

### ePRO platform

The platform used to develop the ePRO system was adapted from the model developed for the eRAPID (Electronic patient self-Reporting of Adverse-events: Patient Information and aDvice) study [30]. The eRAPID IT platform developed at the University of Leeds as an example of a ‘hybrid’ system involving a separate PRO facility linking with an existing EHR. The IT elements, developed in a previous eRAPID study [30] include a patient website (with secure login function), web-based symptom-report questionnaire software (QTool) and a web application interface for secure transfer of the patient data to EHR and viewing of the symptom-report by clinicians. Algorithms can be programmed into the self-report scoring (in QTool), allowing severity-specific tailored self-management advice to be provided to patients and email notifications sent to allocated clinicians. The system was initially developed for patients undergoing systemic therapy and radiotherapy [30–32].

**Fig. 1:**
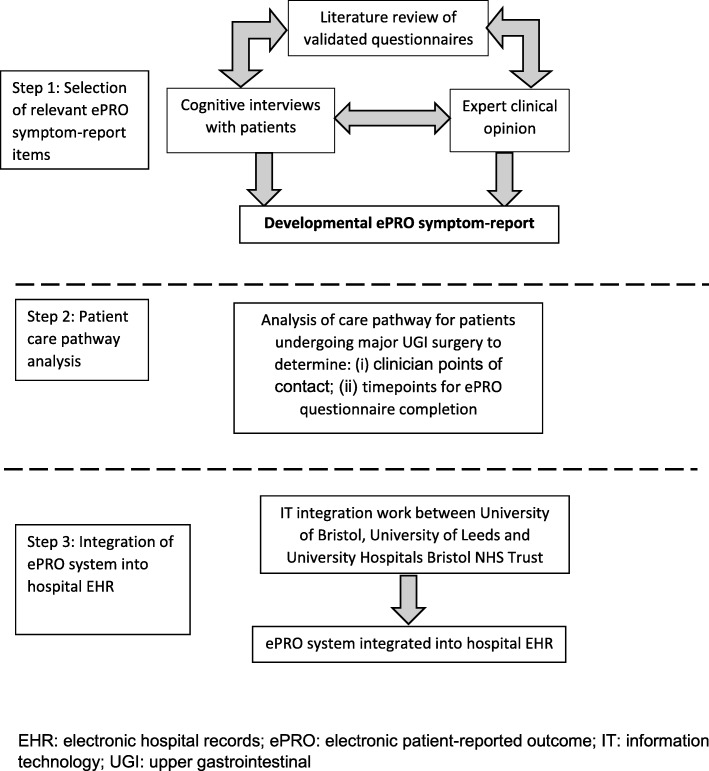
Phase 1: Symptom-report development and ePRO system integration into hospital electronic health records

**Fig. 2:**
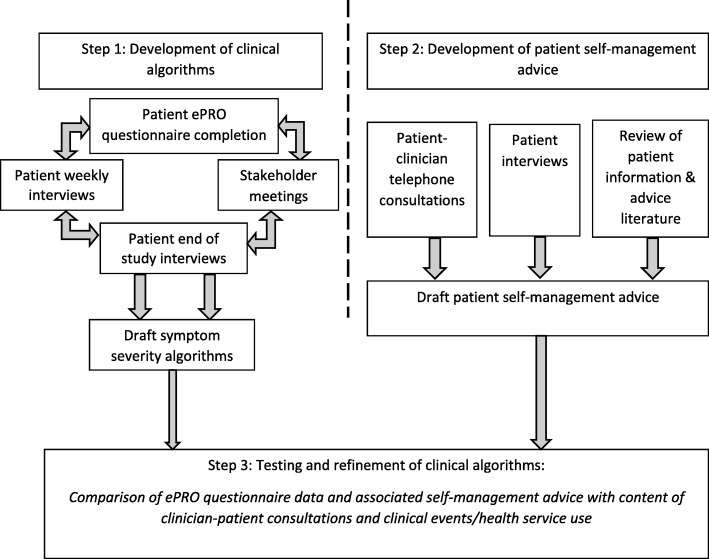
Phase 2: Development and testing of clinical algorithms to guide patient management by symptom severity

The surgical ePRO system was developed between February 2014 and August 2017 in two phases (Figs. 1 and 2), including: (1) Development of the symptom-report for the ePRO system along with necessary IT integration into hospital EHR, and; (2) Development of clinical algorithms to trigger symptom severity-dependent patient advice and clinician alerts based on ePRO self-report responses. Input from key stakeholders, including patients and clinicians, was sought in both phases, as described below.

### Phase 1: symptom-report development and ePRO system integration into hospital EHR (Fig. 1)

#### Step 1: selection of relevant ePRO symptom-report items

Items for inclusion in the ePRO symptom-report questionnaire were identified from a scoping review of existing established, validated European Organisation for Research and Treatment of Cancer (EORTC) questionnaires. EORTC items were selected for their relevance to symptoms and complications experienced by patients after cancer-related major abdominal surgery, including oesophageal, gastric and hepato-pancreato biliary (upper gastrointestinal or UGI) cancer. In this context, UGI is used as a fully inclusive term, to include hepato-pancreato biliary cancer. EORTC items were also selected based on the familiarity of the measures to the clinical and study teams and their established routine use in international trials of PROMs to improve patient outcomes and care [20]. Cognitive interviews with patients with experience of undergoing major UGI surgery were performed to evaluate the acceptability and suitability of the scope of selected items. Between November 2013 and May 2014, inpatient and outpatient lists at University Hospitals Bristol NHS Foundation Trust (UHBT) were screened by a research nurse to identify potentially eligible participants aged 18 or over who had undergone cancer-related major abdominal surgery, including surgery for oesophageal, gastric or hepato-pancreato biliary cancer. Eligible patients were purposively sampled to ensure the inclusion of a wide range of patients. The researcher explained the study and provided the patient with a participant information sheet. The patient was given time to read through the information and ask questions before confirming whether they wished to participate in the study and providing written informed consent. For those patients who expressed an interest in participating, the researcher scheduled a convenient time to conduct the interview either by telephone or face-to-face at the hospital or in the patient’s home. Cognitive interviews were conducted by a researcher to determine participant’s comprehension of the self-report items. Verbal probes were used to investigate participants’ thought processes while responding to items, and participants were encouraged to indicate any trouble understanding or answering items. This work was conducted in iterative cycles of sampling and data analyses to refine the selection of items.

#### Step 2: patient care pathway analysis

Analysis of the care pathway for patients undergoing major UGI surgery at a single UK hospital trust (University Hospitals Bristol NHS Foundation Trust, UHBT) was undertaken by study researchers (EOCF, KA) and the cancer nurse specialist team (JB, TR) to determine relevant clinician points of contact and appropriate timepoints for ePRO self-report completion by patients during their recovery post-discharge following surgery.

#### Step 3: integration of the ePRO system into hospital electronic health records

To facilitate the integration of the ePRO system into the existing hospital EHR system at UHBT (Medway, SystemC), several meetings were held between the study team, the University of Leeds IT team and the UHBT IT team between March 2015 and January 2016. Analysis of IT system performance and integration (e.g. downtime) was undertaken to monitor any issues related to integration.

### Phase 2: development and testing of clinical algorithms to guide patient management by symptom severity (Fig. 2)

Phase 2 of the study was conducted in three steps.

#### Step 1: development of clinical algorithms

Informed by previous research undertaken to develop the eRAPID systems in Leeds and Manchester [32]; [31], it was decided a priori that clinical algorithms would stratify patients into three levels of symptom severity based on their completed ePRO symptom-reports, each triggering a different ‘level’ of action within the ePRO system (Table 1). In addition, so that patients reporting multiple symptoms were not over-burdened with self-management advice, advice was provided for a maximum of the six most clinically concerning symptoms per ePRO self-report completion. To achieve this, four clinicians responsible for the clinical care of patients undergoing major UGI surgery ranked symptoms measured by the ePRO self-report in order of clinical importance. A similar approach has been used in the eRAPID study [30].

**Table 1:** Guided management of patients by symptom severity and ePRO system actions

Symptom severity level	ePRO system action(s)
Level 1: expected symptom(s)	Patient advice: self-management advice
Level 2: potentially concerning symptom(s)	Patient advice: contact a health care professional today if symptom is new or unreported
Level 3: symptom(s) indicative of a complication	(i) Patient advice: contact a health care professional immediately (ii) Clinician alert: automated email to a health care professional

Clinical algorithms to guide severity-specific tailored patient self-management advice based on patient-reported symptoms were developed using four data sources (Fig. 2). Between 15/04/2016 and 15/08/2017, all consecutive patients being discharged from UHBT following cancer-related major abdominal surgery were screened for study eligibility by a cancer nurse specialist at the point of discharge (e.g. immediately preceding or on the day of discharge). Patients were considered eligible to enter the study if they met all the following inclusion criteria: (i) undergone cancer-related major abdominal surgery (including surgery for oesophageal, gastric or hepato-pancreato biliary cancer); (ii) ready for hospital discharge to their home; (iii) have access to personal computer/tablet and internet from home; (iii) sufficient capacity and understanding of English; (iv) aged 18 or over; (v) able to adhere to the study follow-up schedule. Eligible patients were provided with a participant information leaflet explaining the purpose and details of the study when approaching the point of discharge (e.g. on the inpatient ward prior to discharge) and given the opportunity to ask questions by the research nurse. Participants indicating that they wished to participate were asked by the nurse to provide written informed consent and baseline demographic and clinical details were recorded. On the day of discharge, participants were given unique login details and information about accessing the ePRO system via the website on a study information card. A demonstration of the ePRO system was also provided by the research nurse.

Following discharge, participants were requested to prospectively complete the ePRO self-report at discharge (baseline), twice in the first week (day 2 and day 7 approximately) and weekly thereafter for 8 weeks. One email and/or text message reminder with a direct link to the ePRO website was sent to participants on the days they were due to complete the ePRO self-report. However, patients could choose to log in and complete the self-report at any time during this period that they felt unwell or wanted advice. Participants were also asked to participate in a weekly telephone interview with a study researcher/research nurse, in which they were asked to reflect on the nature and severity of any symptoms they had experienced during the previous week. In addition, two stakeholder meetings with a patient and public representative and clinicians responsible for the clinical care of patients undergoing major UGI surgery were conducted. Finally, end of study interviews with a subset of approximately 10% of study participants were conducted approximately 10 weeks post-discharge. Purposive sampling was used to obtain a sample of participants with a diverse range of post-operative experiences and who had used the ePRO system to varying extents. Targeted verbatim transcription of interview audio-recordings was undertaken [33], whereby only those interviews containing data relevant to the research questions were transcribed for analysis (i.e. if a participant’s completion of the ePRO self-report had generated advice or feedback).

Data from the completed patient-completed ePRO self-reports, weekly patient interviews, stakeholder meetings and end of study patient interviews were considered together. Data were prospectively analysed in an iterative, cyclical manner as data collection and analyses proceeded to develop and refine the symptom severity thresholds that would subsequently inform the development of the clinical algorithms to trigger ePRO system actions. The purpose of this was to identify and agree upon symptoms and symptom severities associated with a ‘typical’ recovery following surgery and those that would be considered clinically concerning and to identify symptoms (and associated timepoints) for which advice or reassurance would have been beneficial. These data were used to further refine the selection of items included in the developmental version of the ePRO self-report questionnaire (described above).

#### Step 2: development of patient self-management advice

Patient self-management advice was developed using three data sources. Data were collated from, firstly, audio-recordings of end-of-study interviews (described above) and, secondly, telephone-based clinical consultations undertaken in the first week post-discharge (typically on or around days 2 and 7 post-discharge) between a UHBT cancer nurse specialist (CNS) and participants as part of usual care. These consultations were audio-recorded to explore any advice and/or reassurance offered by the CNS to patients. Targeted verbatim transcription of audio-recordings was undertaken, and data analysed to identify themes relating to post-operative advice and reassurance and consider appropriate phrasing and terminology to inform the development of the ePRO system self-management advice. Between September 2016 and January 2017, a scoping search of online patient information/advice and literature provided by or recommended by the UK National Health Service (NHS) Trusts in England (e.g. on NHS Trust websites or NHS Trust recommended websites such as Macmillan Cancer Support) was undertaken. Search terms specific to patient information and advice (e.g. ‘patient information leaflet’) and relevant surgical procedures (e.g. ‘oesophagectomy’) were used. Information/literature was excluded if it did not relate to relevant procedures or was not from an NHS-approved source. Relevant themes and terminology were extracted from included information/literature using a content analysis approach and used, alongside data from the telephone consultations (described above), to draft the ePRO system patient self-management advice, in accordance with the three pre-determined symptom severity levels described above (Table 1). The draft advice was iteratively refined through discussion within the study group and with clinicians involved in the stakeholder meetings until discussions confirmed that no further iterations were required.

#### Step 3: testing and refinement of clinical algorithms

Data previously generated from a subset of participants’ earlier in Phase 2 of the study (described above) who reported clinically-significant symptoms were reviewed and used to test and refine the clinical algorithms. Participants included in the subset were those who had reported symptoms of a severity that would trigger actions by the ePRO system (i.e. a Level 1, 2 or 3 action). Actions triggered by the ePRO system and the content of any patient self-management advice provided as part of those actions were compared, firstly, with the patient advice provided by the CNS or study research nurse during the routine telephone consultations and weekly telephone interviews and, secondly, with any subsequent clinical events or outcomes of participants (e.g. such as re-intervention, re-admission to hospital, visit to GP or primary healthcare providers). The latter were identified from hospital readmission alerts, hospital EHR, and patients’ reports of accessing healthcare services reported during weekly follow-up telephone interviews. Any discrepancies between actions triggered by the ePRO system and advice provided by the nurses were discussed within the study team. Where considered necessary, the clinical algorithms were refined and tested further in an iterative, cyclical manner until no further refinements were required.

## Results

Sociodemographic and clinical details of participants taking part in Phase 1 and 2 of the study are shown in Table 2.

**Table 2:** Baseline demographic and clinical characteristics of participants

	Phase 1: ePRO self-report item selection interviews (*n* = 18)	Phase 2: ePRO self-report completion participants (*n* = 61)
Sex, n (%)		
Male	16 (89)	35 (57)
Female	2 (11)	26 (43)
Age, years		
Mean (SD)	66.3 (6.7)	61.7 (12.6)
Range	53–80	27–81
Ethnicity^a^, n (%)		
White British	–	46 (75)
Mixed white Asian	–	1 (2)
Not stated	–	14 (23)
Cancer diagnosis, n (%)		
Yes	18 (100)	47 (77)
No	0	14 (23)
Length of hospital stay^a^, days		
Mean (SD)	–	12 (12)
Range	–	2–64
Surgical procedure received^b^, n (%)		
Oesophago-gastric resection	13 (72)	17 (28)
Hepatobiliary resection	5 (28)	44 (72)
Marital status, n (%)		
Married/civil partnership	13 (72)	51 (84)
Single	3 (17)	3 (5)
Divorced	1 (5.5)	4 (6)
Widowed	1 (5.5)	3 (5)
Employment status, n (%)		
Retired	12 (67)	32 (53)
Working full-time	4 (22)	18 (30)
Working part-time	1 (5.5)	4 (6)
Not in paid employment	1 (5.5)	7 (11)

*SD* standard deviation

^a^Data not collected for item selection interview participants

^b^All procedures performed with curative intent

### Phase 1: symptom report development and ePRO system integration into hospital EHR

#### Step 1: selection of relevant self-report items

Ninety-five items from 7 validated EORTC questionnaires were identified. Cognitive interviews with patients (*n* = 18, 16 men, mean age 66 – Table 2) refined the long list of items and confirmed the acceptability and feasibility of items. The final short list of 30 items to be included in the developmental version of the ePRO symptom-report questionnaire were taken from the following cancer-specific health-related quality of life EORTC questionnaires: C30 (core cancer quality of life module), OG25 (oesophago-gastric cancer module), OES18 (oesophageal cancer module), LMC21 (colorectal liver metastases module), and HCC18 (hepatocellular carcinoma module). Consideration by clinical study team members (JB, TR) resulted in 5 additional items not already included in EORTC questionnaires being added (e.g. wound problems), resulting in an ePRO symptom-report comprising 35 items (Table 3).

**Table 3:** Final ePRO self-report items

ePRO self-report items^a^ (*n* = 35)	
1. Did you have any trouble taking a short walk outside of the house?^b^	19. Have you had itching?^b^ (a) Has this itching been due to another condition e.g. dry skin, eczema or allergies?
2. Did you need to stay in bed or a chair during the day?^b^	20. Have you had fevers?^b^ (a) Is this a current issue?
3. Did you need help with eating, dressing, washing yourself or using the toilet?^b^	21. Have you had chills?^b^ (a) Is this a current issue?
4. Were you short of breath?^b^ (a) Have you been short of breath when just sitting down or resting?	22. Have you had pain?^b^
5. Have you had trouble sleeping?^b^	23. Did pain interfere with your daily activities?^b^ (a) Is this a current issue?
6. Have you felt nauseated?^b^ (a) Is your nausea or vomiting stopping you from drinking or eating?	24. Did you need to rest?^b^
7. Have you vomited?^b^ (a) Is your nausea or vomiting stopping you from drinking or eating?	25. Have you felt weak?^b^
8. Have you been constipated?^b^ (a) Are you passing wind?	26. Were you tired?^b^
9. Have you had diarrhoea?^b^ (a) Is this a current issue?	27. Have you lacked appetite?^b^ (a) Is this a current issue?
10. Have you had a dry mouth?^b^	28. Have you had problems eating solid foods?^b^ (a) Could you currently eat solid foods?
11. Have you had trouble with acid or bile coming into your mouth?^b^	29. Have you had problems eating liquidised or soft foods?^b^ (a) Could you currently eat liquidised or soft foods?
12. Have you had acid indigestion or heartburn?^b^	30. Have you had problems drinking liquids?^b^ (a) Could you currently able to drink liquids?
13. Have you had difficulty swallowing your saliva?^b^ (a) Have you been unable to swallow and had to spit out your saliva?	31. Have you had to have any sort of feeding tube fitted to help with nutrition?^c^
14. Have you choked when swallowing?^b^ (a) Is this a current issue?	32. Has your surgical wound been red, warmer than the surrounding skin, swollen or had any leaking fluid?^b^ (a) Is this a current issue?
15. Have you coughed?^b^ (a) Is this a current issue?	33. Has your surgical wound been painful to touch?^b^
16. Have you had abdominal swelling?^b^ (a) Are you passing wind?	34. Have you had any other side effects? (Please state)^c^
17. Have you had a sore mouth or tongue?^b^	35. Have you contacted any health professional regarding any problems? (Please state)^c^
18. Have you been concerned by your skin or eyes being yellow (jaundiced)?^b^ (a) Is this a current issue?	

^a^All ePRO self-report items prefixed with ‘During the past week …’

^b^Response options ‘Not at all’; ‘A little’; ‘Quite a bit’ and ‘Very much’

^c^Response options ‘Yes’ or ‘No’

#### Step 2: patient care pathway analysis

Analysis of patient care pathways identified that the most appropriate time points for ePRO completion were twice in the first week then weekly for 8 weeks post-discharge. The most appropriate clinical contact for patients experiencing potential AEs was identified as the cancer nurse specialist team.

#### Step 3: integration of the ePRO system into hospital electronic health records

Multiple meetings were held with the study and IT team at UHBT between February 2014 and January 2016 to integrate the ePRO system into the UHBT hospital EHR system (Medway). The integrated ePRO system was launched in April 2016. Between April 2016 and August 2017 there were four instances of downtime/integration loss totalling 145 days. This was due to an EHR software update resulting in a loss of EHR integration and clinicians being unable to access ePRO results through the EHR. A temporary workaround solution was developed to allow researchers and clinicians to access ePRO results independently of EHR during periods of downtime. This workaround required clinicians to access the ‘administrator view’ of the live ePRO system hosted on a secure server until an additional software update was issued to resolve the issue.

### Phase 2: development and testing of clinical algorithms to guide patient management by symptom severity

#### Step 1: development of clinical algorithms

**Fig. 3:**
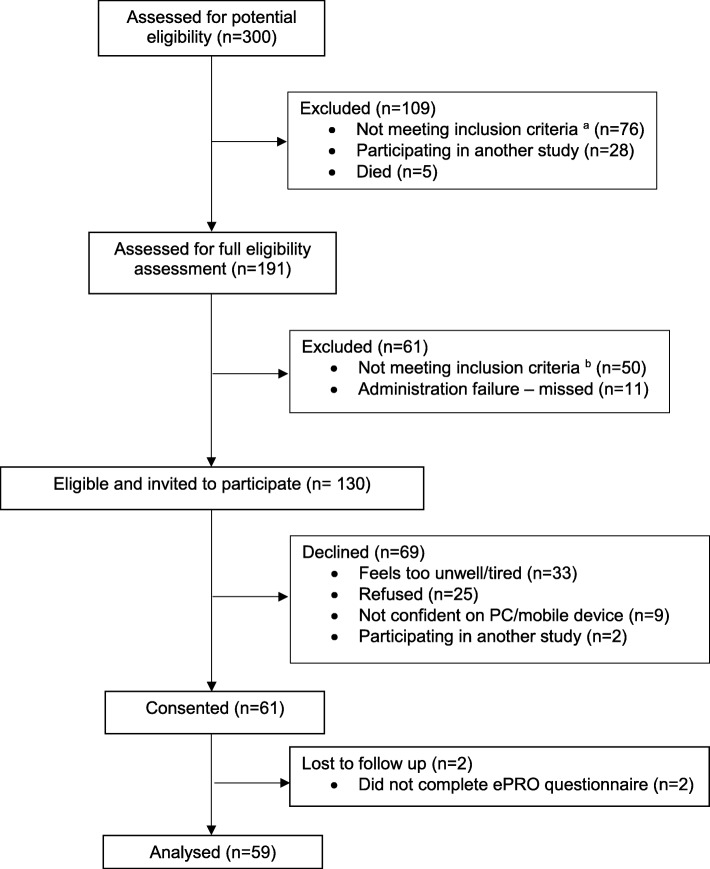
Phase 2 recruitment flow diagram. ^a^ including: *n* = 31 patients not undergoing planned procedure; *n* = 39 patients with no definitive cancer diagnosis (prior to ethics amendment to enable inclusion of patients with no definitive diagnosis); *n* = 5 patients missed due to administration errors; *n* = 1 patient who was under 18. ^b^ including: *n* = 35 patients not having home access to a PC/internet; *n* = 10 patients discharged home unexpectedly early or not discharged to home; *n* = 3 patients not fluent in English; *n* = 2 patients unable to comply with follow up

Of 300 patients screened, 130 (43%) patients were considered eligible and invited to participate, and 61 (47%) patients consented (see Fig. 3). Of these, 59 (97%) participants (34 men, mean age 61 years) accessed the ePRO system a total of 459 times, resulting in 444 completed self-reports. Two (3%) participants (1 man, mean age 72 years) did not complete the self-report at least once and were excluded from subsequent analyses. Analysis of weekly follow-up interviews with these 59 participants and additional end-of-study interviews with 7 participants (4 men, mean age 58 years, described above) indicated a need for additional sub-items and branching logic to make items more relevant to patients’ symptoms and recovery. Specifically, 9 sub-items were added to allow the ePRO self-report to distinguish between ‘typical’ symptoms (e.g. shortness of breath after physical activity) and symptoms indicative of potential AEs (e.g. shortness of breath at rest). Weekly telephone interviews (*n* = 59 participants) also indicated that, for some items, patients tended to report symptoms that were already being appropriately managed or had resolved. To overcome this retrospective reporting of managed or resolved symptoms, an additional sub-item was added to 8 items. This sub-item asked patients if the reported symptom was a current issue, and the response incorporated into the clinical algorithm to trigger the appropriate level of action by the ePRO system in order to guide patient management appropriately.

Two stakeholder meetings (1 patient representative, 6 nurses, 2 dieticians and 1 surgeon from 4 hospital trusts) indicated that, to account for natural improvements in symptoms during recovery, length of time since hospital discharge should be considered alongside symptom severity in the development of the clinical algorithms. For example, a high level of pain is expected at discharge but would be concerning and potentially indicative of an AE if experienced 6 weeks later. The clinical algorithms were refined to incorporate time since hospital discharge accordingly so that threshold scores for relevant symptoms were altered after 3 weeks post discharge to reflect the changes in symptoms experienced during typical recovery. Items included in the final ePRO self-report questionnaire are detailed in Table 3. Details of all changes made to the ePRO self-report during its development are summarised in Table 4.

**Table 4:** Summary of refinements made to ePRO symptom-report and clinical algorithms during ePRO system development

Iterative changes made to ePRO symptom-report		
Area	Issue identified	Changes made
Selection of relevant symptom-report items	Participants reported issues with comprehension and interpretation of some items and response categories.	Initial long list of 95 items from 7 validated EORTC symptom-reports refined to 25 items in accordance with patient interview data.
Selection of relevant symptom-report items	Consultation with clinicians indicated that shortlist of 25 symptom-report items did not cover all symptoms patients are likely to experience following hospital discharge.	Addition of five items, including items about wound problems, feeding tubes, other side effects and any contact with healthcare professionals.
Development of clinical algorithms: *Participant symptom reporting*	Patients could report ‘Very much’ for symptoms that could generate Level 2 or Level 3 actions, but weekly telephone follow-up interviews revealed that symptoms were to be expected depending on context (e.g. shortness of breath after physical activity versus at rest)).	Addition of sub-items and two branching logic questions if potentially concerning symptoms reported: (i) Branching question (“Is this a current issue?”) added to 8 items to determine if reported symptom is a current or resolved problem. (ii) Branching question specific to symptom added to 9 items to clarify severity (e.g. if patient reported ‘quite a bit’ or ‘very much’ shortness of breath, an additional branching question asked, “Were you short of breath while sitting down or resting?”.
Development of clinical algorithms: *Symptom severity thresholds*	Stakeholder meetings and discussion with clinicians indicated that severity of some symptoms is expected to vary during recovery (e.g. high levels of pain are expected during week 1 post-discharge but would be concerning at week 6.	Symptom severity thresholds were adapted for 11 items (e.g. pain, nausea & vomiting, appetite loss) to account for expected variation during recovery between weeks 3–8 post discharge. For example, high levels of pain would generate a maximum of a Level 2 action during weeks 1–2 post-discharge and generate a Level 3 action in week 3.
Development of clinical algorithms: *Combined item scoring*	Patients responses to 1 of the 2 jaundice items (itching) could generate an inappropriate jaundice alert due to the combined scoring of these items, even though itching may be due to allergies, dry skin etc.	Following discussion with clinicians, the scoring for itching and jaundice was split to prevent false positive jaundice alerts. A branching question was also added to the itching item to determine if this was due to a known cause, such as an allergy or dry skin.
Development of patient self-management advice	Patients reported uncertainty about whether their symptoms were expected or concerning and found this troubling.	Following analysis of telephone-based clinical consultations, reassurance relating to expected symptoms was identified and incorporated into Level 1 patient self-management advice.

#### Step 2: development of patient self-management advice

Data from 15 routine care telephone consultations conducted by a CNS with 8 participants (5 men, mean age 62 years) in the first week post-discharge were analysed. Four main themes relating to post-operative advice and reassurance sought by participants and/or provided by the CNS were identified, including pain, other physical symptoms, diet and nutrition and managing recovery (Table 5). Findings from the end of study patient interviews (*n* = 7, 4 men, mean age 58 years) confirmed the acceptability and relevance of self-management advice and reassurance themes. These themes then formed the basis for developing the content of the self-management advice.

**Table 5:** Summary of advice and reassurance themes and subthemes identified from telephone-based clinical consultations during recovery

Theme	Sub-theme	Clinician quotations
Pain	Advice: Pain management using prescribed analgesics and activity levels	*I would think of any medication as a tool there to help you get through something.* *Don’t be surprised if you find that you need them a little bit more than others. If you’re going to do a little bit more then you might find you’ll need to take them.*
	Reassurance: Some level of pain is expected but can be managed	*Surgery can damage the nerve pathways and two weeks afterwards is when they start to mend themselves, so that’s relatively classic that it’s not there immediately after the operation but emerges afterwards.*
Other physical symptoms	Advice: Practical advice relating to food choices, changes in bowel function and ways to manage these	*Just give yourself enough breaks and rests* *Keep in touch with the dietician, keep an eye on the symptoms. One thing you need to keep in mind is that your stomach at the moment is probably the smallest that it will be because it’s still quite swollen so close to the operation.*
	Reassurance: Distinction between expected symptoms and concerning symptoms and how to identify them	*Shortness of breath is a representation of how hard you’re working. If people get short of breath when they’re doing something – that’s part of recovery. What we don’t want to see is someone sat in a chair and having an episode of shortness of breath. But if it’s tied to you exerting yourself that’s part of recovery and will improve with time.*
Diet and nutrition	Advice: The initial approach needs to be flexible and adaptive regarding portion size. Certain food groups need to be avoided	*We want you to tailor our advice to suit you. If you want to eat in stages, that’s perfectly reasonable. You’ll learn to know when you feel full. You’ll learn to know when you’ve had enough and if you leave it half an hour you can come back to it and enjoy round two.*
	Reassurance: Appetite is likely to return and adherence to advice about portion size will improve this	*This is about your appetite and you building it up as you want to and getting you enjoying food again rather than looking at food as a medicine.*
Managing recovery	Advice: Practical advice about pacing physical activities and setting goals	*Our advice [for fatigue] would be ‘do a little, rest a little’ – and the same for shortness of breath.* *Try and be reasonable with your goals but have some goals so at the end of the day you can say ‘yes, I did achieve that, I did make it to the post box and back. A week ago, I was in hospital and now I’m able to walk to the post box and back’*
	Reassurance: Fatigue is expected. Recovery often takes longer than expected and varies day to day	*It’s quite common for people to get that quite marked increase in fatigue when they go home.* *You’re going to have some really good days across those six months and across that year, and there’ll be really good weeks when you think ‘Oh I’m back to normal’ – but then there’ll just be the odd day where you think ‘I have been through a lot, I’m going to take it easy today.*

A total of 28 PILs from 16 NHS Trusts and 3 cancer support charities (Macmillan Cancer Support, Oesophageal Patients Association, Oxfordshire Oesophageal and Stomach Organisation) were sourced. Using methods of content analysis, these data were combined to produce first drafts of patient self-management advice. These first drafts were reviewed and revised according to input from stakeholder meetings (6 nurses, 2 dieticians, 1 surgeon, 1 patient representative), resulting in the development of self-management advice for 22 symptoms. It was recognised through these iterative processes that some symptom advice differed depending on type of surgical procedure; this was made clear in the advice content to ensure patients followed the advice relevant to their procedure.

#### Step 3: testing and refinement of clinical algorithms

Data previously generated from 27 participants’ (18 men, mean age 63 years) earlier in Phase 2 of the study (described above) who reported clinically-significant symptoms were reviewed and used to test and refine the clinical algorithms. Comparisons were made between sets of data (ePRO system actions and advice, clinician telephone advice and clinical events/outcomes) from these 27 patients, and any discrepancies identified and discussed with the study team. Over an 8-month period, refinements to the ePRO self-report questionnaire and clinical algorithms were made (Table 3), including adjustments to the symptom severity thresholds triggering ePRO system actions. Examples of final algorithm thresholds for Level 1, 2 and 3 actions triggered by the ePRO system are shown in Table 6.

**Table 6:** Examples of final algorithm thresholds for Level 1, Level 2 and Level 3 actions

ePRO self-report item question	Item response	Level generated
During the last week, were you short of breath? Sub-item: If yes, have you been short of breath when just sitting down or resting?	Not at all	No feedback
	A little, but not when resting	No feedback
	A little, and while at rest	*Level 2: Advice to contact HCP*
	Quite a bit, but not when resting	Level 1 Self-management advice
	Quite a bit, and while at rest	*Level 2 Advice to contact HCP* ^a^
	Very much, but not when resting	*Level 2 Advice to contact HCP* ^a^
	Very much, and while at rest	Level 3 alert to HCP^b^
During the last week, has your surgical wound been red, warmer that the surrounding skin, swollen or had any leaking fluid? Sub-item: If yes, if this a current issue?	Not at all	No feedback
	A little, but this is not a current issue	No feedback
	A little, and this is a current issue	*Level 2 Advice to contact HCP* ^a^
	Quite a bit, but this is not a current issue	Level 1 Self-management advice
	Quite a bit, and this is a current issue	*Level 2 Advice to contact HCP* ^a^
	Very much, but this is not a current issue	Level 1 Self-management advice
	Very much, and this is a current issue	Level 3 alert to HCP^b^
During the last week, have you vomited? Sub-item: If yes, is your vomiting stopping you from drinking or eating?	Not at all	No feedback
	A little	Level 1 Self-management advice
	Quite a bit, but it has not stopped me from eating or drinking	*Level 2 Advice to contact HCP* ^a^
	Quite a bit, and it has stopped me from eating and drinking	Level 3 alert to HCP^b^
	Very much, but it has not stopped me from eating or drinking	*Level 2 Advice to contact HCP* ^a^
	Very much, and it has stopped me from eating and drinking	Level 3 alert to HCP^b^

^a^Level 2 advises patients to contact a health care professional today if their symptoms are new or unreported

^b^Level 3 advises patients to contact a health care professional immediately. Additionally, an automated email alert is sent to the Cancer Nurse Specialist team

## Discussion

An electronic system for the routine capture of PRO data has been developed to improve the detection and management of complications after hospital discharge following surgery. Unique features of the ePRO system include its full integration into hospital EHR and the function to apply clinical algorithms based on patient-reported symptom-severity to guide the management of patients, and automatically and instantly alert clinicians of atypical symptoms. The ePRO system also accounts for patient-level characteristics to provide individually-tailored self-management advice necessary to provide the wide range of support required by different patients during their recovery at home [34], with patients able to use the system at any time they feel unwell or want advice. In addition, the system is web-based for easy and convenient access by patients using a home computer or portable devices. Full evaluation is now needed to examine its merits compared to standard care.

Until now, few ePRO systems have been developed specifically to support patients after discharge from hospital following surgery. Of 24 controlled trials identified in a 2014 systematic review examining the effects of including PROMs in routine clinical practice [20], only two were relevant to surgical populations [19, 29]. Only one of these was intended for post-discharge follow-up (19). In this study, Cleeland et al. [19] described the use of an ePRO system using interactive voice response technology (as opposed to a web-based system) for triggering clinician alerts to reduce post-operative symptom severity after lung cancer surgery. It is noted that this system did not provide patient self-management advice, was not integrated within patients’ EHR and lacked patient input in the development of alert thresholds [19]. Similarly, Andikyan et al. [26] described evaluation of the feasibility of a web-based ePRO (STAR) system for assessing patient recovery up to 6 weeks after gynaecologic cancer surgery. The STAR questionnaire comprised items from the patient-adaptation of the National Cancer Institute (NCI) Common Terminology Criteria for Adverse Events (CTCAE) and EORTC core questionnaire (QLQ-C30) but was not integrated within hospital EHR and symptom reports were provided to clinicians only at the time of post-operative visits. Embedding ePRO data capture within EHR may be time-consuming and costly but previous research shows that a lack of integration into standard systems is a key barrier to clinicians’ uptake of electronic health systems [35].

This study has several strengths. Multiple data sources, including analyses of interviews and patient-clinician consultations, have directly informed the selection and refinement of ePRO self-report items, content of patient self-management advice and development of the clinical algorithms. The ePRO system has also been developed in close collaboration with multiple key stakeholder groups such as patients, patient representatives, nurses, dieticians and surgeons. This approach has ensured that the ePRO system actions are relevant and meaningful to both patients and clinicians. In addition, the ePRO system uses established, validated measures to assess patients’ HRQL, with the EORTC modules the most frequently used PROMs in oncology studies.

This study does, however, have some limitations. While patient participants with a broad range of sociodemographic characteristics were included, the study was conducted in a single centre in the example context of cancer-related major abdominal surgery and it is possible that the characteristics of the sample do not typify those of the wider population of patients undergoing surgery. There is also a difference in the characteristics of patients who participated in phase 1 and phase 2 of the study, with more patients undergoing oesophago-gastric surgery in phase 1 compared to more undergoing hepatobiliary surgery in phase 2. Further work to develop and test the system in a wider setting and in a broader sample of patients is necessary to evaluate its application as a useful adjunct to patient post-operative care more generally. Depending on the surgical context, the use of alternative disease-specific patient-reported questionnaires may be considered to adapt the ePRO system self-report and algorithms to other patient groups. Integration of the ePRO system with the hospital EPR was lost for a total of 145 days over the 17-month study period due to an unsupported EHR software update. While it was possible to implement a temporary workaround, this incident highlights the potential impact IT issues can have on the widespread use of ePRO systems within EHR and indicates that successful use in routine clinical care would require full integration into hospital EHR and ongoing maintenance by hospital IT departments.

Surgery is associated with significant complications and AEs, many of which occur after patients are discharged home and require further clinical intervention. Self-management of symptoms in the absence of adequate medical knowledge to distinguish between expected and concerning symptoms can be worrying and burdensome for patients and delay necessary treatment [7–9]. An ePRO system with the functionality to apply clinical algorithms to automatically alert clinicians in real-time of atypical symptoms has the potential to improve symptom control, patient safety and outcomes and decrease emergency admissions, with the additional potential of healthcare cost savings. Findings from a randomised evaluation of the STAR ePRO system by Basch et al. showed that the routine and real-time reporting of PROs significantly reduces hospital readmissions and improves patients’ survival and HRQL in cancer patients compared to usual care, though this single centre trial had several methodological weaknesses and limited generalisability [15]. Another RCT concluded that automated symptom monitoring via an interactive voice response system and clinician alerts reduced symptom severity in 100 patients during the month after lung cancer surgery compared to automated monitoring plus usual care alone [19]. Symptoms were assessed using a validated though generic cancer PROM. The study was also small and conducted in a specialist tertiary care centre, limiting its generalisability. It is also unclear how patients were selected for inclusion in the study. Furthermore, neither of these systems provided feedback or advice to patients for symptom self-management or were integrated in routine EHR.

## Conclusions

Embedding prospectively-collected ePRO data into routine clinical practice has the potential to bring wider benefits to patients and healthcare systems through standardising practice [13], streamlining and enhancing clinical consultations [20, 25] and optimising personalised and patient-centred care [24]. Data collected may also be valuable to informing NHS policy and the development of future treatments and services. Nevertheless, standardised and routine ePRO data capture in patients discharged from hospital after surgery is lacking. This study describes the development of a real-time electronic symptom monitoring system for patients after discharge following cancer-related surgery. A multicentre prospective pilot study is ongoing to fully evaluate the usability and acceptability of the ePRO system, including data completeness, profiles of patient-reported symptoms and actions triggered, and patients’ and clinicians’ experiences of its usefulness. This pilot work will inform a future RCT that will compare the effectiveness and cost-effectiveness of the ePRO system versus usual care for improving the detection of symptoms, complications and patient outcomes after hospital discharge following major elective surgery.

## Abbreviations

AE: Adverse effect
EHR: Electronic health record(s)
ePRO: Electronic patient reported outcome
eRAPID: Electronic patient self-Reporting of Adverse-events: Patient Information and aDvice
ERAS: Enhanced recovery after surgery
HCP: Healthcare professional
HRQL: Health-related quality of life
PRO: Patient-reported outcome
PROM: Patient-reported outcome measure
UGI: Upper gastrointestinal
